# Symptoms of exhibitionism that regress with bupropion: A case report

**DOI:** 10.3389/fpsyt.2022.1079863

**Published:** 2023-01-04

**Authors:** Sefa Vayısoğlu

**Affiliations:** Independent Researcher, Adana, Turkey

**Keywords:** exhibitionistic disorder, treatment, bupropion, serotonergic antidepressants, dopaminergic activity

## Abstract

Exhibitionistic Disorder, one of the paraphilic disorders, is a disease with an unknown etiology and causes significant distress and loss of function in the patient's life. Serotonergic antidepressants are generally preferred in the treatment of this Disorder. However, in this case, we report a patient who did not respond to serotonergic antidepressants but bupropion, an antidepressant with dopaminergic and noradrenergic activity. Therefore, bupropion should be considered a medical treatment alternative in case serotonergic antidepressants do not work efficiently in the treatment of Exhibitionism.

## Introduction

Exhibitionism, one of the paraphilic disorders according to DSM-5, is a disease characterized by repetitive, sexually stimulating, intense fantasies, urges, or behaviors related to displaying the genitals to a stranger who does not expect such action for at least 6 months. It causes clinically substantial discomfort or function distortion in social, occupational, educational, or personal areas (1).

Paraphilic disorders and hypersexual behavior can be examined as behavioral disorders in the possible categorization of impulsivity and compulsivity endophenotypes as impulsivity-compulsivity disorders as a transdiagnostic psychopathological dimension together with Obsession Compulsion Related Spectrum Disorders, Substance/Behavioral Addictions, Disruptive/Impulsive Behavior Disorders. In response to the question of why impulsivity and compulsions cannot be stopped in various psychiatric disorders, it is stated that it may be due to a problem in the cortical circuits that suppress these behaviors. Impulsivity and compulsivity are hypothetically “bottom-up” neurobiological impulses, impulsivity originates from the ventral striatum, compulsivity from the dorsal striatum, and different areas of the prefrontal cortex try to suppress these stimuli “top-down” (2).

There have been case reports and studies conducted on clomipramine (3–5), fluoxetine (6, 7), fluvoxamine (8), paroxetine (9), and trazodone (10), which are antidepressants with significant serotonergic effects that are reported to be effective in the pharmacological treatment of this disorder.

A patient with Exhibitionistic Disorders did not react to serotonergic antidepressants, escitalopram, paroxetine, and sertraline but did respond to bupropion, a noradrenaline and dopamine reuptake inhibitor was addressed in this case report. However, there are no examples of Exhibitionistic Disorders responding to bupropion treatment in the literature, within the knowledge of the author of this case report.

## Case

A 33-year-old male patient applied to the psychiatry outpatient clinic with his wife. As a complaint, he stated that he had painful thoughts and sexual impulses of unexpectedly revealing his genitals to persons he did not know in public, which he had difficulty resisting. He claimed that this propensity became apparent in his adolescence, but it was not to a degree of frequency that bothered him until 3 years ago. He stated that his sexually stimulating fantasies and impulses had risen significantly, virtually every day and throughout the last 3 years. At first, he preferred adult women, but then he also started tending to adult males. He admitted to three failed attempts and felt a strong feeling of remorse. He had become less social and presented social withdrawal during these 3 years. Both the patient and his wife expressed concern that he would lose his will and act, that he would get into legal problems due to these thoughts and desires, and that he would be introduced to the media. His wife corroborated her spouse, who explained that he has been married to his wife for 6 years, has a daughter, and has a regular sexual life. The frequency of sexual intercourse, which was three times per week in the first 2 years of their marriage, has dropped to one per week in the last 2 years. The patient's wife stated they had satisfactory intercourse and said she could not understand how such a feeling and behavior developed. The patient claimed that the sexual arousal he had as a result of his thoughts and drives connected to the exhibitionism was far more intense and pleasurable than the traditional sexual intercourse he experienced with his wife and that masturbating soothed him whenever he thought he was about to lose control and take action. When his symptoms began to worsen 3 years ago, he received psychotherapy for 6 months under the supervision and follow-up of a clinical psychologist. However, it did not work, so he began therapy sessions with another psychologist. He stated that there was no substantial change in him. He maintained therapy for 6 months before quitting due to financial reasons. He claimed that he suspected he had excessive testosterone hormone at the time due to the research he did on the internet, so he went to a private laboratory and had a test, but his testosterone level was average. He admitted that, despite being started on escitalopram 10 mg/day by his psychiatrist about 2 years ago, then increased to 20 mg/day a month later and used in this manner for about 6 months, there was no change in his impulses and fantasies, and he had difficulty falling asleep at night for the last 2 years; in addition, there were not any hypomanic symptoms or anything else suggestive of an attenuated mixed mood state accompanying this trouble in falling asleep noted by the patient and his wife. He said another psychiatrist he consulted ceased using escitalopram and started paroxetine 20 mg/day and trazodone 50 mg/day for sleep. He noted that trazodone helped his sleep. Despite the paroxetine 20 mg/day he was taking, there was no apparent reduction in his urges and thoughts regarding exhibitionism. He stated that in the 3-month follow-up, the dose of paroxetine was increased to 30 mg/day, and he used paroxetine at an amount of 30 mg/day for 3 months. When there was no significant effect, the dose of paroxetine was increased to 40 mg/day, but he could only use it for 3 days, and it caused nausea, dizziness, and heart palpitations. Following that, he stated that paroxetine was progressively reduced and terminated by the psychiatrist within a week, and he started taking sertraline 50 mg/day. He noted that the dose of sertraline was increased up to 200 mg/day at the end of 4 months with 1-month control. He was taking this dose for about a year, and he simultaneously started psychotherapy by a psychologist under the guidance of his psychiatrist during this period. The frequency of his impulses and fancies decreased from 7 days to 4–5 days per week. However, there was no substantial reduction in the intensity of his whims and fantasies and, therefore, the distress he caused himself.

His medical history did not reveal any remarkable features. He was not a smoker. He did not do any drugs. He said that his alcohol intake was at the level of social drinking, consisting of beer or a single raki with friends. However, he did not consume alcohol during this period since he had not participated in social activities for the previous year. In his family history, he stated that his mother had been using escitalopram for years and that the reason was unknown, but he suspected it was for depressive symptoms. The patient, who worked as a technical staff in a company, stated that he had begun to have mild concentration problems in the last few months, that he sometimes could not perceive what was said, that he forgot immediately even if he perceived it at the time and that both his boss and his colleagues noticed this.

His mood was mildly depressed, and his affection was slightly enhanced in the direction of sorrow, according to his mental state evaluation. Thoughts of guilt and concern about legal issues that could occur in the future predominated his thoughts. No indications of any personality disorders were detected. There was no evidence of perceptual deviation. His orientation was complete, and his memory test results were typical. There was no evidence of a judgment problem, and his understanding was perfect. The total blood count, vitamin B12 level, thyroid function tests, and liver and kidney findings were all within the normal reference range.

Given that the patient had not benefited from the previous selective serotonin reuptake inhibitors (escitalopram, paroxetine, sertraline), it was decided to begin bupropion 150 mg/day, which is from a different group with dopaminergic and noradrenergic effects, rather than trying another antidepressant from the same group. Sertraline was progressively reduced and terminated within a week, while trazodone was maintained in the evening for sleep. After 1 month, the patient said that the intensity and frequency of his fantasies and impulses connected to exhibitionism had diminished substantially. As a result, he felt much better morally. His inability to concentrate and distraction had vanished as if he had been suffering from a shortage of bupropion in his brain for years. When his deficit was remedied, he returned to his senses. One month later, the patient who quit taking trazodone was examined in the outpatient clinic control. He also claimed that his sleep returned to normal and that he had a few days of shallow sleep after quitting the trazodone but that his sleep pattern returned to normal in the following days. The patient was found to be in good health during the third control of the outpatient clinic in the sixth month of follow-up and was recommended to complete the medication therapy for 1 year.

## Discussion

The exact etiology of paraphilia and paraphilic disorders is unknown. However, it is suggested that a combination of neurobiological, interpersonal, and cognitive processes may be involved. In Impulsive and Compulsive Disorders, including paraphilic disorders, neuroanatomically impulsivity and compulsivity map to different neuronal loops: impulsivity is a ventral striatum-dependent action-outcome learning system, whereas compulsivity is a dorsal striatum-dependent habituation system. Stimuli initiate many behaviors in the ventral loop of motivation and reward. Over time, some of these behaviors migrate to the dorsal region through a series of neuroadaptations and neuroplasticity and become habitual, meaning that impulsive behavior eventually becomes compulsive. These information spirals, which pass from one neuron cycle to another, receive regulatory inputs from the hippocampus, amygdala and other prefrontal cortex areas. Low-dose slow-release stimulants may improve people's ability to say no to an impulsive request by increasing dopamine and decreasing impulsivity in the Orbitofrontal Cortex (OFC) circuit, which is part of the prefrontal cortex (11). In our case, it was observed that the impulsive tendencies of the person decreased, and his willpower strengthened with the slow-release form of bupropion.

Dopamine may have a significant role in paraphilic disorders, according to a recent study focused on the function of neuronal transmission in these diseases. The study's researchers point out that a decrease in the activity of the dopaminergic system probably causes stereotyping of behavior and determines the psychopathological peculiarity of paraphilic localization (12). In our case, the patient with exhibitionism, a paraphilic disorder, did not react to serotonergic antidepressants but did respond to bupropion, which has a substantial dopaminergic impact, implies that the neurobiological basis of the disease may get connected to the dopamine system.

Bupropion is an antidepressant that influences noradrenaline and dopamine activity but has little impact on serotonin. Despite case reports of individuals with Exhibitionism Disorder responding to serotonergic medications (3–10), the underlying pathogenic mechanism in some instances, such as ours, may be connected to other neurotransmitters (dopamine and noradrenaline) rather than serotonin.

Although serotonergic monotherapies (Selective Serotonin Reuptake Inhibitors or Tricyclic antidepressants, mainly tertiary amines such as clomipramine, amitriptyline, and imipramine) are indicated as viable alternatives in nonviolent paraphilic disorders (13), antidepressants that primarily operate on the dopaminergic and noradrenergic systems should not be disregarded.

Further clinical research is required to explain the neurobiological mechanisms behind paraphilic disorders in general and Exhibitionistic Disorders in particular and to develop treatments.

## Data availability statement

The original contributions presented in the study are included in the article/supplementary material, further inquiries can be directed to the corresponding author.

## Ethics statement

Ethical review and approval was not required for the study on human participants in accordance with the local legislation and institutional requirements. The patients/participants provided their written informed consent to participate in this study. Written informed consent was obtained from the individual(s) for the publication of any potentially identifiable images or data included in this article.

## Author contributions

The author confirms being the sole contributor of this work and has approved it for publication.

## References

[1] American Psychiatric Association. Diagnostic and Statistical Manual of Mental Disorders, Fifth Edition, Text Revision. Washington, DC: American Psychiatric Association (2013), p. 689. 10.1176/appi.books.9780890425596

[2] Stahl SM. Stahl'in Temel Psikofarmakolojisi, Sinirbilimsel Temeli ve Pratik Uygulamasi, Transl. by T. Alkin. Istanbul: Istanbul Medikal Saglik ve Yayincilik (2015), p. 539–41.

[3] Wawrose FE Sisto TM. Clomipramine and a case of exhibitionism. Am J Psychiatry. (1992) 149:843. 10.1176/ajp.149.6.843a 1590505

[4] Casals-Ariet C Cullen K. Exhibitionism treated with clomipramine. Am J Psychiatry. (1993) 150:1273–4. 10.1176/ajp.150.8.1273b 8328583

[5] Torres AR Cerqueira AT. Exhibitionism treated with clomipramine. Am J Psychiatry. (1993) 150:1274. 10.1176/ajp.150.8.1274c 8369077

[6] Perilstein RD Lipper S Friedman LJ. Three cases of paraphilias responsive to fluoxetine treatment. J Clin Psychiatry. (1991) 52:169–70. 2016251

[7] Bianchi MD. Fluoxetine treatment of exhibitionism. Am J Psychiatry. (1990) 147:1089–90. 10.1176/ajp.147.8.1089c 2375447

[8] Zohar J Kaplan Z Benjamin J. Compulsive exhibitionism successfully treated with fluvoxamine: a controlled case study. J Clin Psychiatry. (1994) 55:86–8. 8071253

[9] Abouesh A Clayton A. Compulsive voyeurism and exhibitionism: a clinical response to paroxetine. Arch Sex Behav. (1999) 28:23–30. 10.1023/A:1018737504537 10097802

[10] Terao T Nakamura J. Exhibitionism, and low-dose trazodone treatment. Hum Psychopharmacol Clin Exp. (2000) 15:347–9. 10.1002/1099-1077(200007)15:5<347::AID-HUP203>3.0.CO;2-S 12404312

[11] Stahl SM. Stahl'in Temel Psikofarmakolojisi, Sinirbilimsel Temeli ve Pratik Uygulamasi, Transl. by T. Alkin. Istanbul: Istanbul Medikal Saglik ve Yayincilik (2015), p. 573.

[12] Kamenskov MY Gurina OI. Neurotransmitter mechanisms of paraphilic disorders. Zh Nevrol Psikhiatr Im S S Korsakova. (2019) 119:61–7. 10.17116/jnevro201911908161 31626172

[13] Guay DR. Drug treatment of paraphilic and nonparaphilic sexual disorders. Clin Ther. (2009) 31:1–31. 10.1016/j.clinthera.2009.01.009 19243704

